# Effectiveness of A Pilates Training Program on Cognitive and Functional Abilities in Postmenopausal Women

**DOI:** 10.3390/ijerph17103580

**Published:** 2020-05-20

**Authors:** Patricia Alexandra García-Garro, Fidel Hita-Contreras, Antonio Martínez-Amat, Alexander Achalandabaso-Ochoa, José Daniel Jiménez-García, David Cruz-Díaz, Agustín Aibar-Almazán

**Affiliations:** 1 Department of Health Sciences, Faculty of Health Sciences, University of Jaén, 23071 Jaén, Spain; vidaymovimiento2012@hotmail.com (P.A.G.-G.); fhita@ujaen.es (F.H.-C.); amamat@ujaen.es (A.M.-A.); dcruz@ujaen.es (D.C.-D.); aaibar@ujaen.es (A.A.-A.); 2 Department of Teaching Physical Education, Fine Arts and Music, University of Cádiz, 11003 Cádiz, Spain; josedanieljimenezgarcia@gmail.com

**Keywords:** cognitive performance, functional skills, older women, Pilates

## Abstract

The purpose of this study was to determine the effects of a Pilates exercises program on the cognitive and physical functioning of older Spanish women. This study is a randomized clinical trial; a total of 110 women aged ≥60 years were initially allocated to either a Pilates group (PG, n = 55), who underwent a 12-week Pilates exercise program, or to a control group (CG, n = 55), who did not receive any intervention. Global cognitive function (Mini-Mental State Examination), verbal fluency (Isaacs test), executive function (Trail Making Test), functional flexibility (Back Scratch Test and Chair Sit-and-Reach Test), and lower-body strength (30 s Chair-Stand Test) were assessed before and immediately after the intervention period. The main findings of this study suggest that women in the PG (within-group differences) experienced improvements across all the variables examined except for global cognitive function. When compared with the CG (between-group differences), our analysis revealed significant benefits in the PG for all measures except for global cognitive function and functional flexibility (Back Scratch Test). In conclusion, our results suggest that Pilates has the potential to improve both cognitive and functional abilities among Spanish women aged 60 years and over.

## 1. Introduction

The aging population is experiencing rapid growth as a result of improvements in medical care and standard of living [1]. Worldwide, those over 60 years of age amount to 11% of the population—percentage that is estimated to increase to 22% by 2050 [2], posing significant challenges to healthcare systems all over the world in the coming decades.

Several functional changes have been related to the aging process, and cognition is a crucial aspect of aging. Cognitive aging, which can affect diverse cognitive functions such as memory, processing speed or understanding, is considered a public health concern [3]. Lower cognitive function and cognitive decline has been associated with decreased functional capacity regarding daily-life activities, disability, poor quality of life, and increased risk of mortality [4,5].

Physical exercise is widely recommended for older women to maintain health and functional capacity [6]. In recent studies, physical exercise programs have been shown to improve the basic and instrumental activities of daily life among older women [7,8,9]. Similarly, physical exercise plays a fundamental role in protecting against cognitive decline [10], as it induces structural and functional changes in the brain that provide considerable biological and psychological benefits [11].

Recently, new physical training programs based on mind–body exercises have been suggested for the prevention of cognitive decline in older people [12,13]. The Pilates method is a combination of strength, flexibility, and balance exercises. It focuses on lumbo-pelvic stabilization, with the activation of the deep muscles of the trunk, and seeks a complete connection of body and mind [14]. This training system is not limited to any age group, and has in fact been widely recommended to older adults given its lack of impact exercises [15]. The Pilates method has been shown to have beneficial effects on both physical and psychological health [16,17]. However, randomized clinical trials testing the effects of cognition-related Pilates exercises on healthy older women are sparse [18].

In accordance with the above considerations, the objective of this study is to determine the effects of a Pilates training program on the cognitive and physical functioning of women aged 60 years and over. We hypothesize that Pilates exercises are able to improve their global cognitive function, verbal fluency, timed motor and visual tasks, functional flexibility, and lower-body strength.

## 2. Materials and Methods

### 2.1. Study Design

This was a randomized controlled trial which aimed to analyze the effects of a 12-week Pilates intervention on the cognition and functional abilities of postmenopausal Spanish women (https://clinicaltrials.gov/ct2/show/NCT03201107). Participants registered in July 2017 and the intervention was carried out between September and December 2017. Participants signed an informed consent form before the start of the study, which was approved by the Human Ethics Committee of the University of Jaén (JUN.17/1), and performed in accordance with the Declaration of Helsinki, Good Clinical Practice, and all applicable laws and regulations.

### 2.2. Participants

**Figure 1:**
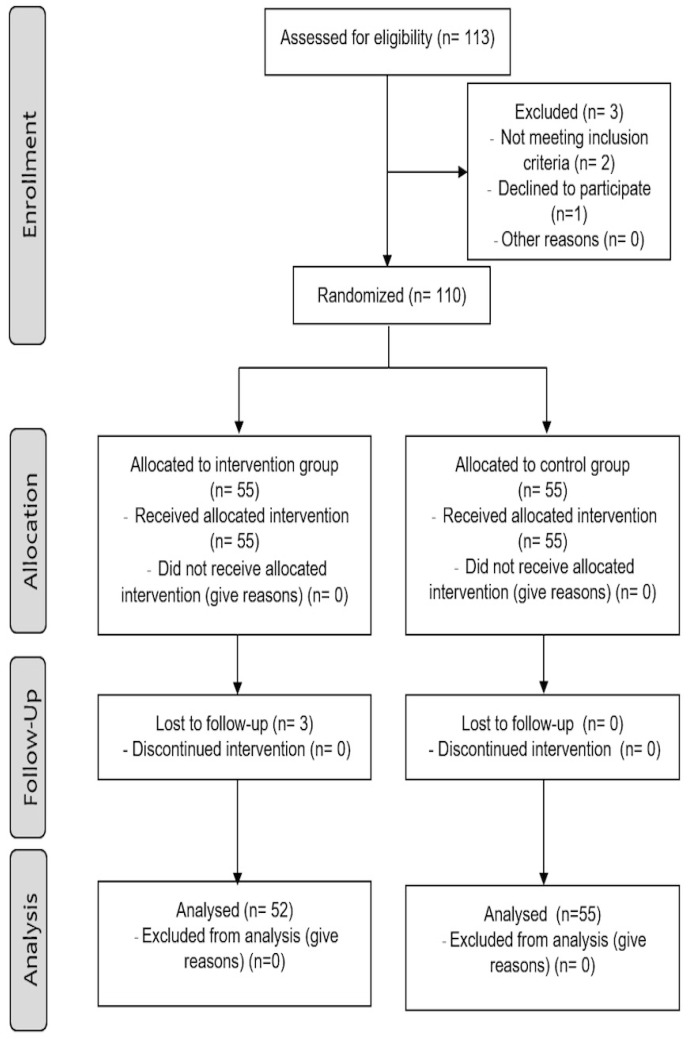
Flow diagram of study design.

Recruitment took place through two associations of postmenopausal women in Jaén (Spain). Participants were contacted via email or telephone. Out of 113 women who were initially evaluated for inclusion in the study, 110 met the inclusion criteria and agreed to be included (Figure 1). Women who (i) were 60 years old and over, (ii) had not participated in a Pilates training program in the previous 12 months, and (iii) were physically independent to perform basic daily activities were included in the study. Prospective participants were excluded when they suffered from any condition that prevented them from exercising or were already enrolled in a different training program.

### 2.3. Randomization

Participants were assigned using a computer-generated table of random numbers to either a Pilates group (PG) or a control group (CG) in a proportion of 1:1. All participants, researchers, and the physiotherapist in charge of administering the intervention were blinded to group assignment. Assignments were made using sealed opaque envelopes, consecutively numbered and kept under lock, which were opened by an independent administrator. Finally, 55 subjects were assigned to the PG and 55 to the CG.

### 2.4. Intervention

Participants included in the PG performed a clinical Pilates exercises program, which, compared to traditional Pilates, are designed by a qualified physical therapist and adapted to the study population, guaranteeing the safety of performing an exercise without risk. The Pilates exercise program comprised two sessions per week over a 12-week period. Each session consisted of a 10 min warm-up, a 35 min main program, and a 15 min cool-down, for a total of 60 min. For the warm-up and cool-down routines, static stretching exercises were performed to increase muscle relaxation. As for the main program, the exercises were divided into four parts and required a basic–intermediate level of effort. The first session provided the required familiarization with the Pilates method and its principles, the correct execution of movements, and proper breathing. The second part comprised a week of chair-based exercises. During the third part, floor-based exercises were carried out for four weeks. Finally, during the last six weeks, participants employed a series of implements such as elastic bands, magic rings, and balls. All sessions were supervised by a qualified and experienced professional. No injuries or adverse effects were observed or reported during the intervention period. A minimum attendance to 80% of the training sessions was required for inclusion in the analysis. Participants assigned to the control group simply maintained their daily lifestyle, received a set of guidelines aimed at encouraging physical activity, and were prohibited from participating in any formal training. They were periodically contacted by telephone during the 12 weeks of intervention period and questioned about their physical activity habits.

### 2.5. Outcomes

All outcomes were collected before and just after the end of the intervention period. Descriptive characteristics such as age, level of education, and marital and occupational status were collected before allocation by means of self-administered questionnaires in the presence of experienced interviewers. Independent researchers blinded to group allocation performed the outcomes assessment. Height and weight were assessed by a T201-T4 Asimed adult height scale and a 100 g–130 kg precision digital weight scale (Tefal), respectively. Body mass index (BMI) was calculated as weight (kg)/height (m^2^) [19].

#### 2.5.1. Cognitive Function

The Mini-Mental State Examination (MMSE) is one of the most widely used tests to measure global cognitive function and is designed to detect serious cognitive impairments. We used the 30-item Spanish version [20], which explores five cognitive areas: orientation, registration, attention and calculation, recall, and language. The maximum score is 30 points. Higher scores indicate better overall cognition on the part of the subject. Reference scores are as follows: normal, 27 or more; pathological suspicion, 24 or less; deterioration, 12 to 24; and dementia, 9 to 12.

#### 2.5.2. Verbal Fluency

The Isaacs test provides a measure of verbal fluency. Participants have 60 s to generate as many words as possible within a given semantic category (animals, colors, fruits, and cities). The maximum score is 40 points (with a maximum of 10 per category). The higher the score, the better the level of verbal fluency of the subject. Semantic memory refers to the memory used for generic, over-learned information, including memory for word names [21].

#### 2.5.3. Executive Function

The Trail Making Test (TMT) [22] was used to assess executive function. It measures timed motor and visual tasks, and it is divided into two tests: part A (TMTA), which evaluates speed and psychomotor attention and requires connecting consecutively numbered circles; and part B (TMTB), which evaluates executive function and requires connecting alternating circles of numbers and letters. Longer completion times indicate poor performance.

#### 2.5.4. Functional Flexibility

In order to evaluate functional flexibility, we used the Back Scratch Test (BST) [23] for both upper extremities, and the Chair Sit-and-Reach Test (CSRT) [24] for both lower extremities. The BST was used to assess shoulder joint flexibility. It was performed in the standing position, placing one hand behind the neck and moving it down the spine. Meanwhile, the other hand was placed in the lower back and moved up the spine. The procedure was repeated with opposite arms/hands. The distance between the tips of the middle fingers of both hands was measured; if the fingers only touched, “zero” was marked. If the fingers did not touch each other, the distance (cm) was measured in negative values (−). If the fingers of the hands overlapped, the distance was recorded in positive values (+). The CSRT was used to evaluate lower-body flexibility—mainly the hamstrings. Participants sat in a chair placed against a wall for stability purposes, and tried to touch the toes of both their right and left feet. If the toes were only contacted by the fingers, the score was “zero”. Reaches short of the toes were recorded in negative (−) values, with positive (+) values assigned to those able to reach beyond (in cm).

#### 2.5.5. Lower-Body Strength

The 30 s Chair-Stand Test (30s-CST) [25] was used to assess lower-body strength. Participants sat with their back in the upright position in a chair without armrests and their arms crossed in front of their chest [26]. They had to stand up and sit down as many times as possible within 30 s. A higher number of repetitions indicated greater lower-body strength.

### 2.6. Sample Size Calculation

The sample size was calculated using Ene 3.0 (GlaxoSmithKline, SA, Madrid, Spain). The required sample was determined by taking as a reference the data reported by Nishiguchi et al. [27]. To obtain a statistically significant difference using MMSE scores as the dependent variable, with a power of 0.80, a significance level of 95%, and considering an estimated drop out of 30%, a minimum of 39 subjects per group were required.

### 2.7. Statistical Analysis

Mean values and standard deviations for each variable of interest were calculated. The Student’s t test for independent samples was used to examine the differences between both study groups. A mixed variance analysis was employed in which the intervention (PG vs. CG) was the between-group factor and the time of measurement (pre-treatment vs. post-treatment) was the within-subject variable. Dependent variables were MMSE, Isaacs Test, TMT (parts A and B), BST (left and right arms), CSRT (left and right legs), and 30s-CST. Separate analyses were performed for each dependent variable. A possible interaction between treatment (group) and measurement time was examined. Cohen’s *d* was used to calculate intergroup effect sizes, where a value of ≤0.2 indicated a small effect, 0.5 a medium effect, and 0.8 a large effect [28]. A *p* value below 0.05 was considered statistically significant. Statistical analyses were performed using the SPSS statistical software, version 17.0 (SPSS, Inc., Chicago, IL, USA).

## 3. Results

Descriptive information of the participants at baseline is displayed in Table 1. At that point, our analysis did not reveal any significant differences between both groups. All subjects took part in at least 91.6% of sessions, and no injuries or adverse effects were reported over the course of the intervention. None of the participants had a MMSE score ≤ 24, which indicated suspected cognitive impairment (values ranged from 25–30).

### 3.1. Global Cognitive Function

MMSE scores did not reveal any significant main effects regarding the variables group, time, and the interaction of group × time (Table 2).

### 3.2. Verbal Fluency

Table 2 shows the main effects for verbal fluency. According to our findings, higher (better) scores were obtained in the Isaacs test after the Pilates training program: t (54) = −10.46, *p* < 0.001, Cohen’s *d* = 0.70. A between-group comparison of the scores shows that, after the intervention, the values reached by the PG were higher than those of the CG: t (105) = −2.21, *p* = 0.029, Cohen’s *d* = 0.43.

### 3.3. Motor and Visual Tasks

As far as motor and visual tasks are concerned (Table 2), values observed in the PG immediately after the Pilates intervention were significantly lower than previous measurements (and therefore indicative of improved performance): t (54) = 8.57, *p* < 0.001, Cohen’s *d* = 0.47. Furthermore, between-group differences could be observed after the intervention: t (105) = 4.41, *p* < 0.001, Cohen’s *d* = 0.85. As for TMTB, the analysis revealed significant differences in the PG between values taken before and after the intervention: t (54) = 9.71, *p* < 0.001, Cohen’s *d* = 0.45. Post-intervention values in the PG were also significantly better than in the CG: t (105) = 4.80, *p* < 0.001, Cohen’s *d* = 0.93.

### 3.4. Functional Flexibility

Our analysis showed that participants in the PG saw their BST and CSRT results improve after the intervention period (Table 3). As for the BST, significant within-group differences were found in the PG for both the right-arm: t (54) = −5.16, *p* < 0.001, Cohen’s *d* = 0.15, and the left-arm tests: t (54) = −6.99, *p* < 0.001, Cohen’s *d* = 0.11. A study of between-group differences after the intervention yielded improved results for right-arm BST: t (105) = −2.21, *p* = 0.029, Cohen’s *d* = 0.42. No significant differences were found between both groups in measurements taken after the intervention period.

CSRT results showed significant within-group differences, with women who underwent the Pilates intervention exhibiting improved outcomes in both the right leg—t (54) = −8.86, *p* < 0.001, Cohen’s *d* = 0.58—and left leg—t (54) = −7.95, *p* < 0.001, Cohen’s *d* = 0.41. The postintervention analysis revealed between-group improvements in the PG for both right-leg CSRT—t (105) = −4.02, *p* < 0.001, Cohen’s *d* = 0.77—and left-leg CSRT—t (105) = −3.69, *p* < 0.001, Cohen’s *d* = 0.71.

### 3.5. Lower-Body Strength

Finally, an analysis of the 30s-CST results (Table 3) showed that participants in the PG scored significantly higher (better) after the intervention period: t (54) = −8.80, *p* < 0.001, Cohen’s *d* = 0.68. Moreover, between-group differences were significant in measurements taken after the intervention: t (105) = −6.03, *p* < 0.001, Cohen’s *d* = 1.17.

## 4. Discussion

The aim of the present study was to assess the effects of a 12-week Pilates intervention on the cognitive and physical functioning of women aged 60 years and over. The findings showed that verbal fluency, executive function, lower-body strength and functional flexibility improved after a training program of Pilates exercises, although no significant between-group differences in functional flexibility were observed after the intervention period.

Cognitive deterioration has become one of the greatest challenges facing healthcare systems, families and communities, and the importance of the prevention and early treatment of conditions such as dementia is ever increasing [29]. In the present study, participants who received a Pilates-based training showed no improvement in global cognition after 12 weeks. In agreement with our results, a previous randomized controlled trial (RCT) carried out among older adults of both sexes [30] found that a training program focused on strength, flexibility, and balance exercises did not influence cognitive function. On the other hand, Jurakic et al. [31] found a beneficial effect on global cognition, using the Montreal Cognitive Assessment tool (MoCA), after an 8-week Pilates training program. However, and unlike our work, their study involved elderly women with mild cognitive impairment. One possible explanation for such disparity is that participants in our study scored higher in the MMSE because they did not experience any cognitive impairment, and that any improvement they experienced was therefore not significant.

Language functions such as verbal fluency, verbal recovery, and naming tend to decline with age [32]. Our findings revealed that a 12-week Pilates training program improved verbal fluency as measured by the Isaacs test. Along the same lines, several studies have reported improvements in verbal fluency after different types of physical training. One such study conducted a randomized controlled trial in a population of older people and reported improvements in measures of word list fluency after a training program that combined flexibility, aerobic, resistance, and balance exercises for 13 months [33]. Another study [34] found improvements in verbal fluency among older adult, following an aerobic exercise-based intervention that lasted 12 weeks. However, Lam et al. [35] reported that a training program comprising stretching, body and mind (Tai Chi), and aerobic exercises had no benefits for verbal fluency in older people with mild cognitive impairment. This may be due to the fact that language functions tend to remain in a better condition among healthy people than in those who experience worsened deterioration during the aging process. The present study contributes new data concerning the effects of other types of training such as Pilates on the verbal fluency of older women, a decidedly growing and at-risk population.

Executive functions involve advanced cognitive processes which are deemed necessary for behavioral control in humans [36]. In the present RCT, the TMT was used to assess executive function, and improvements were found in the group that performed Pilates training. As far as we know, this is the first study linking a Pilates program with executive function in older women. Despite there being some previous studies that link this function with other types of training, their results were unremarkable. For instance, Ansai et al. [37] reported no improvements in executive functions among older adults after 16 weeks of training involving aerobic, strength, and balance exercises. In the same way, Kimura et al. [38] also failed to find statistically significant improvements in executive function after 12 weeks of strength training in older people. The fact that our results are so dissimilar may be due to the fact that women practicing Pilates must remember new movement patterns and sequences. The frontal lobe may therefore be involved in the regulation of executive functions required to complete tasks in a Pilates training [39]. Some laudable hypotheses can be used to explain these benefits on cognitive functions. It has been reported that physical exercise has neuroprotective and neuroplastic effects on brain structures [40]. Besides, Pilates exercises emphasize the coordination of body movements and rhythmic breathing, as well as the connection between the body and the mind [41]. The characteristics of this exercise method have been determined to be associated with increased hippocampal volumes and stimulation of the frontal lobes—aspects that play a major role in preserving the cognitive functions studied.

Similarly, and in addition to the beneficial effects of physical exercise on cognitive abilities, it has been demonstrated that regular practice of physical exercise improves function among older women. Flexibility plays a fundamental role in the functional capacity of the elderly, in their performance of daily activities, and in their achievement of functional autonomy [42]. Traditional exercise training programs, such as resistance exercises, have been shown to have positive effects on functional flexibility among older women [43], and a double-blind randomized trial that compared the effects of three types of training (aquatic, aerobic, and strength exercises) on older women concluded that the three programs resulted in improvements in flexibility, but that strength training did so to a greater degree and in a shorter time [9]. In recent years, some new types of exercise and training programs have been the subject of research. In a randomized controlled trial carried out by Im et al. [44], elderly women were randomly assigned to a combined Korean dance and yoga exercise program for 12 weeks. They found that, compared to a control group, women assigned to yoga saw improvements in their flexibility as measured by CSRT. In the same way, Zou et al. [45] found a beneficial effect on flexibility after 8 weeks of Yang-style Tai Chi among healthy older women. As for Pilates exercises, Curi et al. [46] reported improvements in flexibility after 16 weeks of a Pilates exercise intervention in women aged ≥60 years. The findings of the present RCT show that women enrolled in a 12-week Pilates training program experienced an improvement in functional flexibility after the intervention period when compared with a control group.

Decreased muscle strength and flexibility affect the functional performance of tasks that require motor coordination and balance, including life activities. This hinders a healthy lifestyle, which is why it has been argued that exercise is the basis for the improvement of functional skills in older women [47]. A systematic review supported the beneficial effects of Pilates training on the physical fitness, balance, and fall prevention in 60–80 year-old women and yielded significant improvements in their lower-body strength [48]. Our findings revealed that a Pilates training program brought about considerable improvements in lower-body strength as assessed by the 30s-CST, with significant post-intervention intergroup differences. Similar results have been previously found among women aged 60 years and above after a Pilates training, but only in longer intervention programs such as the study by Curi et al. [46], which lasted 16 weeks, and that of Plachy et al. [49], which took 24 weeks. Furthermore, authors such as Bergamin et al. [15] reported in a pilot study that Pilates exercises were able to improve muscle strength in older women. However, the design of their study (quasi-experimental) did not allow for comparisons with a control group. In general, the results of our RCT on functional capacities indicate that, for women aged ≥60 years, Pilates can be considered to improve flexibility and strength, which are linked to balance. Therefore, from a clinical perspective, Pilates could be useful to reduce the risk of falls and improve the independence and quality of life of women [50].

Among the strengths of our study, we may mention its randomized, blinded, controlled trial design, its high rate of adherence to the interventions and its large sample size. However, this study also has some limitations. Firstly, only short-term effects were evaluated. In addition, the present study was conducted among women living in a community, and its conclusions cannot be extended to every population. Future studies should be carried out considering medium and long-term effects in both older men and women.

## 5. Conclusions

The present study, conducted in a population of older women, shows that a Pilates training program of 12 weeks (with twice-weekly sessions) has beneficial effects on cognitive abilities such as verbal fluency and executive function. In addition, improvements in the functional abilities of functional flexibility and lower-body strength were found. These results have considerable clinical implications for the target population, since they open new possibilities of delaying cognitive and functional deterioration, therefore improving the autonomy and quality of life of older women.

## Figures and Tables

**Table 1:** Baseline characteristics of the study group.

		Total (n = 107)	CG (n = 52)	PG (n = 55)	*p*-Value
Age		68.18 ± 8.35	66.79 ± 10.14	69.98 ± 7.83	0.070
Weight (kg)		70.16 ± 11.10	68.60 ± 10.27	71.63 ± 11.73	0.160
Height (cm)		1.55 ± 0.06	155.31 ± 0.06	154.07 ± 0.07	0.293
BMI		29.40 ± 4.54	28.62 ± 4.44	30.17 ± 4.55	0.074
Occupational Status Marital Status Education	Retired	70 (65.42)	32 (61.54)	38 (39.09)	0.597 0.849 0.113
	Worker	15 (14.02)	9 (17.31)	6 (10.91)	
	Unemployed	22 (20.56)	11 (21.15)	11 (20.00)	
	Single	3 (2.80)	1 (1.92)	2 (3.64)	
	Married	52 (48.59)	25 (48.08)	27 (49.09)	
	Divorced/Widow	52 (48.59)	26 (50.00)	26 (47.27)	
	Nothing	22 (22.43)	8 (15.38)	16 (29.09)	
	Primary	47 (43.92)	21 (40.38)	26 (47.27)	
	Secondary	26 (24.30)	17 (32.69)	9 (16.36)	
	University	10 (9.35)	6 (11.54)	4 (7.27)	
MMSE		28.07 ± 1.78	28.31 ± 1.70	27.85 ± 1.84	0.189
Isaacs test		29.06 ± 8.13	30.46 ± 8.61	27.73 ± 7.50	0.082
TMTA		146.64 ± 84.15	148.67 ± 89.38	144.73 ± 79.66	0.810
BST-right		231.93 ± 72.91	230.62 ± 74.73	233.18 ± 71.82	0.857
TMTB		−12.55 ± 16.06	−14.38 ± 19.54	−10.82 ± 11.82	0.253
BST-left		−16.80 ± 18.40	−18.31 ± 21.47	−15.38 ± 15.01	0.414
CSRT-right		−4.56 ± 8.83	−3.29 ± 8.49	−5.76 ± 9.05	0.148
CSRT-left		−6.42 ± 10.41	−6.81 ± 11.24	−6.05 ± 9.65	0.710
30s-CST		12.64 ± 4.01	12.37 ± 4.11	12.91 ± 3.94	0.486

The quantitative variables are presented as mean ± standard deviation. Qualitative variables are presented as frequency (percentage); BMI, Body Mass Index; MMSE, Mini Mental State Examination; TMTA, the Trail Making Test—Part A; TMTB, the Trail Making Test—Part B; BST, Back Scratch Test; CSRT, Chair Sit and Reach Test; 30s-CST, 30 s Chair-Stand Test.

**Table 2:** Effects of Pilates training on global cognitive function, verbal fluency, and motor and visual tasks.

	Preintervention		Postintervention		Group			Time			Group × Time		
	CG	PG	CG	PG	F (1.80)	*p*	η^2^	F (1.80)	*p*	η^2^	F (1.80)	*p*	η^2^
MMSE	28.31 ± 1.70	27.85 ± 1.84	28.23 ± 1.76	27.91 ± 1.80	0.29	0.259	0.01	0.10	0.756	0.00	3.34	0.071	0.03
Isaacs test	30.46 ± 27.73	27.73 ± 7.50	29.12 ± 9.44	32.49 ± 6.06	0.05	0.833	0.00	37.18	<0.001	0.26	118.82	<0.001	0.26
TMT-A	148.67 ± 89.38	144.73 ± 79.66	176.02 ± 89.10	110.80 ± 62.22	5.37	0.022	0.05	0.53	0.467	0.01	46.11	<0.001	0.31
TMT-B	230.62 ± 74.73	233.18 ± 71.82	261.56 ± 586.46	200.56 ± 73.43	4.98	0.028	0.05	0.07	0.791	0.00	101.94	<0.001	0.50

Variables are presented as mean ± standard deviation. PG, Pilates Group; CG, Control Group; MMSE, Mini Mental State Examination; TMTA, the Trail Making Test—Part A; TMTB, the Trail Making Test—Part B.

**Table 3:** Effects of Pilates training on functional flexibility and lower-body strength.

	Preintervention		Postintervention		Group			Time			Group × Time		
	CG	PG	CG	PG	F (1.80)	*p*	η^2^	F (1.80)	*p*	η^2^	F (1.80)	*p*	η^2^
BST-right	−14.38 ± 19.54	−10.82 ± 11.82	−16.17 ± 20.55	−9.09 ± 11.53	2.88	0.093	0.03	0.01	0.918	0.000	34.98	<0.001	0.25
BST-left	−18.31 ± 21.47	−15.38 ± 15.01	−19.52 ± 1.46	−13.78 ± 14.71	1.49	0.225	0.01	0.36	0.247	0.01	71.14	<0.001	0.40
CSRT-right	−3.29 ± 8.49	−5.76 ± 9.05	−7.94 ± 10.38	−0.75 ± 8.05	1.99	0.161	0.07	0.14	0.714	0.001	95.45	<0.001	0.48
CSRT-left	−6.81 ± 11.24	−6.05 ± 9.65	−9.67 ± 1.71	−2.29 ± 8.90	4.20	0.043	0.04	1.78	0.186	0.02	96.66	<0.001	0.48
30s-CST	12.37 ± 4.11	12.91 ± 3.94	11.02 ± 3.91	15.60 ± 3.94	11.85	0.001	0.10	12.45	0.001	0.12	112.24	<0.001	0.52

Variables are presented as mean ± standard deviation. PG, Pilates Group; CG, Control Group; BST, Back Scratch Test; CSRT, Chair Sit and Reach Test; 30s-CST, 30 s Chair-Stand Test.
